# Multiple paraovarian cysts requiring emergency surgery: A rare clinical finding

**DOI:** 10.1002/ccr3.5321

**Published:** 2022-02-06

**Authors:** Marie Tominaga, Kyoko Morikawa, Yutaro Ogawa, Hiromi Ishiguro, Naomi Kamimura, Tomokazu Yokoo, Ikunosuke Tsuneki, Masaki Tamura, Toru Yanase, Takumi Kurabayashi

**Affiliations:** ^1^ 26834 Department of Obstetrics & Gynecology Niigata City General Hospital Niigata Japan

**Keywords:** laparoscopy, mature cystic teratoma, ovarian cyst, paraovarian cyst, torsion

## Abstract

This report presents an unusual case of multiple paraovarian cysts that required emergency surgery due to a paraovarian cyst being entrapped by another paraovarian cyst. Laparoscopic surgery is considered useful for diagnostic and therapeutic purposes and is, therefore, recommended owing to difficulty in differentiating paraovarian cysts from ovarian cysts.

A 38‐year‐old woman was brought to our hospital with left lower abdominal pain. She had been diagnosed with a left ovarian cyst approximately 10 years ago and had undergone observation at another hospital.

An emergency computed tomography (CT) scan revealed a 55‐mm ovarian cyst with suspected torsion of the left ovary tube (Figure 1). Laparoscopic examination revealed enlargement of the left ovary with no evidence of torsion. There were four paraovarian cysts near the left fallopian tube with a cyst entrapped by another that appeared necrotic.

**FIGURE 1:**
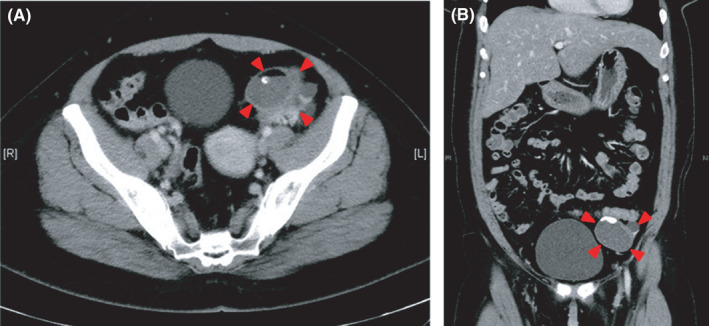
Computed tomography imaging findings. (A) Transverse computed tomography (CT) and (B) coronal CT images demonstrate 55‐mm cystic lesions with calcification and fluid formation in the left ovary (red arrowheads). The presence of torsion is unknown

The ovarian and paraovarian cysts were removed (Figure 2A–D). The pathological findings revealed the ovarian cysts to be benign mature cystic teratomas, and the paraneoplastic ovarian cysts to be benign serous cysts (Figure 3). The postoperative course was uneventful, with no recurrence.

**FIGURE 2:**
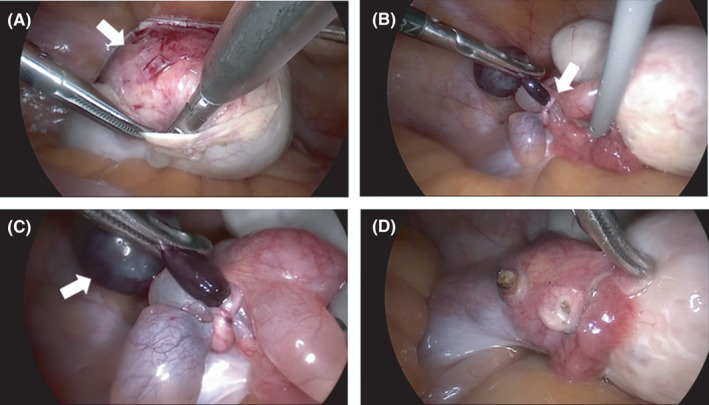
Intraoperative photographs. (A) Image of an ovarian cyst being enucleated. The left ovary is enlarged to 5–6 cm (white arrow) with no evidence of torsion. (B) (Far view image) There are four paraovarian cysts near the left fallopian tube, one of which is entrapped by another (white arrow). (C) (Near view image) The entrapped paraovarian cyst has a purple discoloration and is suspected to be necrotic (white arrow). The remaining paraneoplastic ovarian cysts and ovarian cysts do not show torsion. (D) The ovarian and paraovarian cysts are removed

**FIGURE 3:**
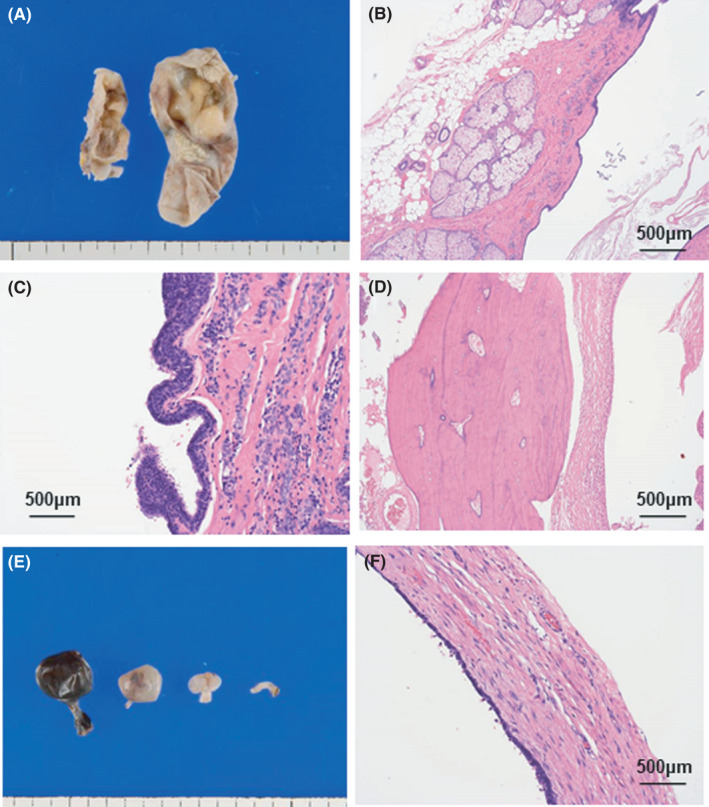
Macroscopic and pathological findings. (A) Macroscopic findings of ovarian cysts. (B‐D) Pathological examination shows mature cystic teratoma (skin appendages, adipose tissue, airway epithelium, and bone tissue are present). (E) Macroscopic findings of paraovarian cysts. (F) Pathological examination shows cysts lined by serous epithelium. No malignant characteristics were found

Paraovarian/paratubal cysts constitute about 10% of adnexal masses that are usually asymptomatic and rarely cause torsion.^1^ These cysts have no pedicle, and when they cause torsion, the ovary, fallopian tube, and infundibulopelvic ligament are often involved.^2^

This is the first report of entrapment of a paraovarian cyst by another. Preoperative diagnosis was challenging; however, laparoscopy was useful for diagnostic and therapeutic purposes.

## CONFLICT OF INTEREST

The authors declare that they have no current financial arrangement or affiliation with any organization that may have a direct influence on their work.

## AUTHOR CONTRIBUTIONS

All the authors made substantial contribution to the preparation of this manuscript and approved the final version for submission. MT, KM, YO: drafted the manuscript; MT: is the corresponding author; HI, NK, TY, IT, MT and TY: provided clinical support; TK: carefully reviewed the manuscript.

## CONSENT

Written informed consent was obtained from the patients for the publication of their information and imaging.

## Data Availability

Not applicable.
